# Perinatal Distress in Women in Low- and Middle-Income Countries: Allostatic Load as a Framework to Examine the Effect of Perinatal Distress on Preterm Birth and Infant Health

**DOI:** 10.1007/s10995-014-1479-y

**Published:** 2014-04-20

**Authors:** Shahirose Premji

**Affiliations:** 1 Faculty of Nursing, University of Calgary, 2500 University Drive NW, Calgary, AB T2N 1N4 Canada; 2 Department of Community Health Sciences, Faculty of Medicine, University of Calgary, TRW Building, 3rd Floor, 3280 Hospital Drive NW, Calgary, AB T2N 4Z6 Canada; 3 Alberta Children’s Hospital Research Institute, Heritage Medical Research Building, 3300 Hospital Drive NW, Calgary, AB T2N 1N4 Canada

**Keywords:** Maternal stress, Pregnancy outcome, Infant, preterm, Allostatic load, Developing countries

## Abstract

In low- and middle-income countries (LMIC), determinants of women’s and children’s health are complex and differential vulnerability may exist to risk factors of perinatal distress and preterm birth. We examined the contribution of maternal perinatal distress on preterm birth and infant health in terms of infant survival and mother–infant interaction. A critical narrative and interpretive literature review was conducted. Peer-reviewed electronic databases (MEDLINE, Embase, Global Health, CINHAL), grey literature, and reference lists were searched, followed by a consultation exercise. The literature was predominantly from high-income countries. We identify determinants of perinatal distress and explicate changes in the hypothalamic–pituitary–adrenal axis, sympathetic, immune and cardiovascular systems, and behavioral responses resulting in pathophysiological effects. We suggest cultural–neutral composite measures of allostatic mediators (i.e., several biomarkers) of maternal perinatal distress as objective indicators of dysregulation in body systems in pregnant women in LMIC. Understanding causal links of maternal perinatal distress to preterm birth in women in LMIC should be a priority. The roles of allostasis and allostatic load are considered within the context of the health of pregnant women and fetuses/newborns in LMIC with emphasis on identifying objective indicators of the level of perinatal distress and protective factors or processes contributing to resilience while facing toxic stress. We propose a prospective study design with multiple measures across pregnancy and postpartum requiring complex statistical modeling. Building research capacity through partnering researchers in high-income countries and LMIC and reflecting on unique ethical challenges will be important to generating new knowledge in LMIC.

## Introduction

Both perinatal distress and preterm birth are world-wide problems that are especially burdensome in low- and middle- income countries (LMIC). Maternal prenatal and postnatal distress (i.e., stress, anxiety, or depression at any time in pregnancy and during the first year following birth of the infant), collectively referred to as “perinatal distress,” may be significantly higher in LMIC than high income countries [1]. The prevalence of perinatal mental disorders reported for LMIC is comparable to certain high-risk groups of women living in high-income countries [1–3]. In LMIC the determinants of women’s and children’s health are complex. Moreover, inequities in determinants of health and the social, cultural, and political contexts of women in LMIC negatively influence women’s mental health. Consequently, differential vulnerability may exist not only to risk factors of perinatal distress, but also to predictors of pregnancy outcome [4].

Stress, anxiety, or depression during pregnancy may contribute to preterm birth [5, 6]. Every year, 15 million babies are born prematurely, and 1.1 million will die due to prematurity-related health issues globally [7, 8]. Twelve of the 15 countries which contribute more than 60 % to the global burden of preterm birth are low or low-middle income countries [9]. Preterm birth is one of the major contributors to infant mortality and morbidity [7, 10], accounting for 80 % of the world’s 1.1 million deaths [9]. Africa and South Asia, with the exception of Pakistan, have made some progress in improving neonatal survival; however, death resulting from preterm birth is now the second leading cause of newborn deaths [7, 9]. Up to 50 % of pediatric neurodevelopment problems (e.g., cerebral palsy, lower intelligence quotient) are estimated to be the result of preterm birth [11–14].

Perinatal distress may also adversely influence infant survival, behavior, and development through poor quality of maternal–infant interactions [15–21]. A Taiwanese population-based study, that linked birth and death certificate registry, found the adjusted risk of mortality among preschool children up to age 5 years was 1.47 fold (95 % Confidence Interval, CI 1.16–1.87) when mothers experienced depression in the first year following birth [22]. Infants born in LMIC are already exposed to poverty, poor health, and poor nutrition, which reduces their developmental potential [23]. Beyond these issues, infants of depressed mothers are less likely to be breastfed, have incomplete immunizations, have poorer weight gain, and are more likely to experience illnesses, such as diarrhea, which in turn, may increase the number of hospital admissions and contribute to higher mortality in children under 5 years of age [24–31].

Clinicians typically rely on self-report questionnaires to assess perinatal distress. While very useful, self-report is prone to bias or error [32]. An alternative is to use biomarkers that may offer a more objective and quantifiable indicator of the level of perinatal distress [33]. The conceptual framework of allostatic load [20] links perinatal distress and its physiological responses to multisystem dysregulation, which promotes a cascade of events ultimately impacting pregnancy outcome (i.e., preterm birth) and infant health (i.e., survival and development) [16, 18–20, 34]. In this context, biomarkers that detect physiological compromise may be useful predictors of perinatal distress and its negative consequences. Specifically, perinatal distress may activate aspects of the hypothalamic–pituitary–adrenal (HPA) axis, sympathetic, immune and cardiovascular systems, and promote behavior changes (e.g., smoking, drinking) in the effort to restore allostasis [13, 20]. Over time, given “wear and tear” on the brain and body, biological responses may be compromised, or fail outright. Allostasis refers to the continual changes in set points (i.e., lower or higher ranges) of physiologic systems to maintain constancy [20, 34] with repeated and ongoing (i.e., chronic) exposure to determinants of perinatal distress over the course of pregnancy [16, 35, 36]. The resulting dysregulation of interrelated systems may, over time, reach a “tipping-point” [16] referred to as allostatic load or overload, that ultimately results in pathophysiological effects. In the case of perinatal distress, effects can include preterm birth [20, 34] and altered maternal and infant behaviors that adversely influence infant survival and development [15, 16, 18–21, 37].

A critical narrative and interpretive review [38] was undertaken to: (a) determine the etiologic contribution of perinatal distress on preterm birth in pregnant women in LMIC; and (b) develop a conceptual framework that would explicate the potential casual links of perinatal distress to preterm birth and infant health (i.e., infant survival, and mother–infant interaction). The goal of the review was to inform future research in LMIC by providing a conceptual framework to examine psychosocial and environmental factors as both risk factors and targets of intervention to prevent preterm birth (i.e., improve maternal health outcomes) and improve infant survival and development.

## Methods

### Search and Selection Strategy

**Fig. 1:**
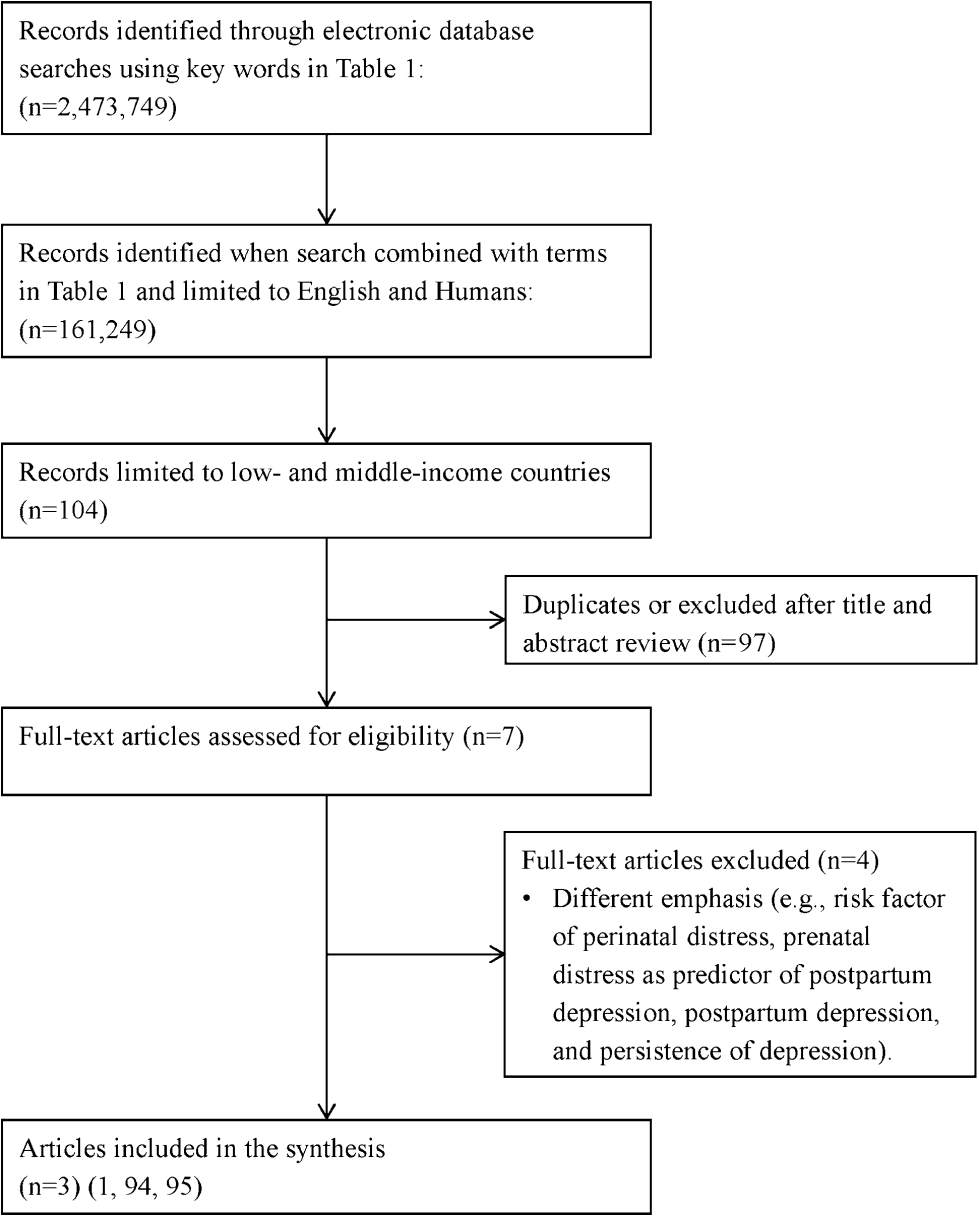
Overview of trial flow through the search and selection process: Contribution of perinatal distress on preterm birth

**Fig. 2:**
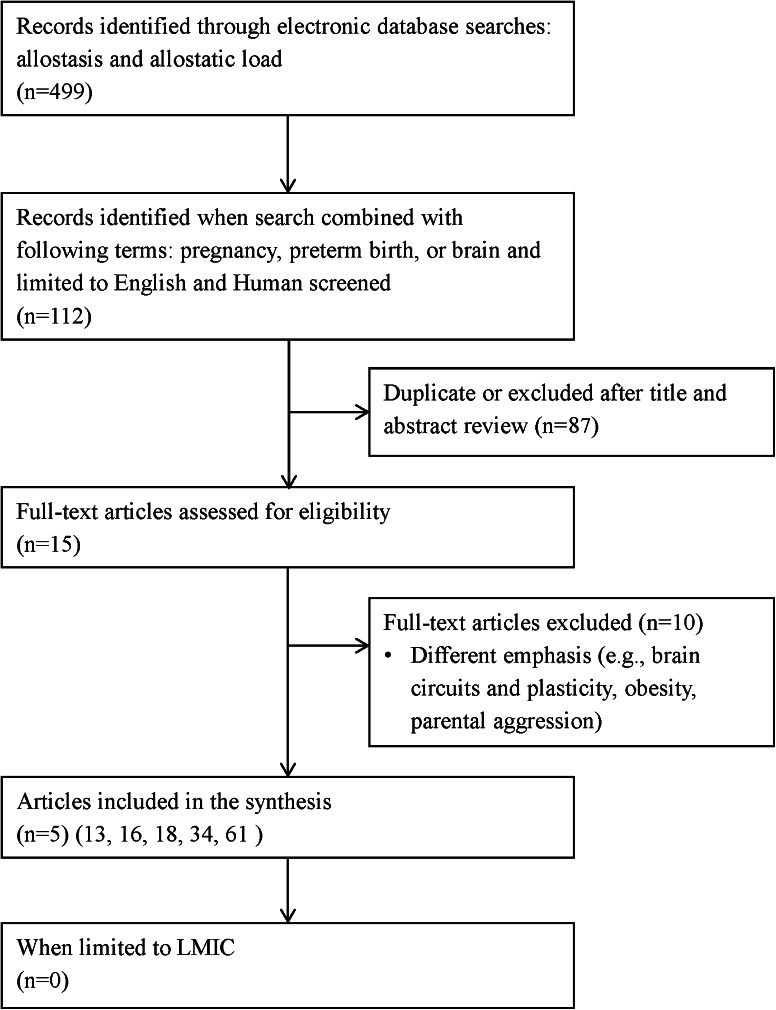
Overview of trial flow through the search and selection process: Allostasis and allostatic load

We searched peer-reviewed electronic databases including MEDLINE (1946–January 2013), Embase (1974–January 2013), Global Health (1910–January 2013), and Cumulative Index to Nursing and Allied Health Literature (CINHAL) (1990–January 2013). Grey literature (e.g., unpublished theses, organizational websites), reference lists, and an existing network of experts in the area (including research team members from Pakistan, Kenya, and Tanzania) were also used in identifying relevant publications. A conventional review technique using the search strategy and selection strategy detailed in Table 1 proved to be limiting given the dearth of literature in LMIC (see Figs. 1, 2). In contrast, a search of the existing literature using all key words, combined terms, and exclusion criteria (i.e., etiology and conceptual framework) without limiting the country of origin generated 6,908 records.

**Table 1:** Key words, combined terms, and selection criteria

	Key words	Combined terms	Selection criteria
Etiologic contribution of perinatal distress on preterm birth	Stress; anxiety; depression; stress, maternal; stress, psychological; chronic stress; postpartum depression; perinatal distress; or perinatal depression	Pregnancy; women; pregnant, women; perinatal outcomes; infant health; infant survival; mother–infant interaction; maternal health; or preterm birth	Pregnant or postpartum women; recruitment in low- and middle-income countries; assessed psychosocial health/factors; examined any determinant of health that would impact maternal psychosocial well-being and maternal or infant health; any study design; human; English
Conceptual framework	Allostasis or allostatic load	Pregnancy; preterm birth; or brain	Pregnant or postpartum women; recruitment in low- and middle-income countries; maternal health; infant health; any study design; human; English

Applying a precise review question or narrowing the search by assembling certain levels of evidence is restrictive when the intent of the literature review is also to generate a theory [38]. Consequently, we used a critical narrative and interpretive synthesis approach [38], based in dialectic process including both inductive and deductive reasoning, to guide our sampling of the extant literature, regardless of study type and location of study, while maintaining a focus on the aims of the review. As a starting point we used our earlier review [39] on the relationship between prenatal stress, depression, cortisol and preterm birth, and the literature reviewed here. We then purposefully sampled the existing literature to elaborate on the phenomena of interest and our analysis of the literature. The approach we used to develop the conceptual framework was iterative and the emphasis of the review changed and was informed by our emerging understanding and analysis of the literature (i.e., recursive and reflexive). We continued to sample the literature until there was saturation, that is, similar ideas emerged repeatedly [38]. A total of 73 articles identified through this iterative process complemented the eight articles identified in the initial search (see Figs. 1, 2).

### Quality Assessment and Data Extraction

All types of studies were valued for their contribution, as they provided new ways of understanding our emergent conceptual framework and causal links between perinatal distress and preterm birth. Criteria for assessment included: (1) whether the study design was appropriate given the aim and objectives of the study; (2) appraisal of study reporting (e.g., data collection process described, appropriate method of analysis, enough data shared to support interpretation and conclusions); or (3) judgment about whether the study clarified what is known and what is not known, and informed the interpretation of concepts or the review in general [38]. No papers were removed because of poor methodological standards.

### Consultation Exercise

**Fig. 3:**
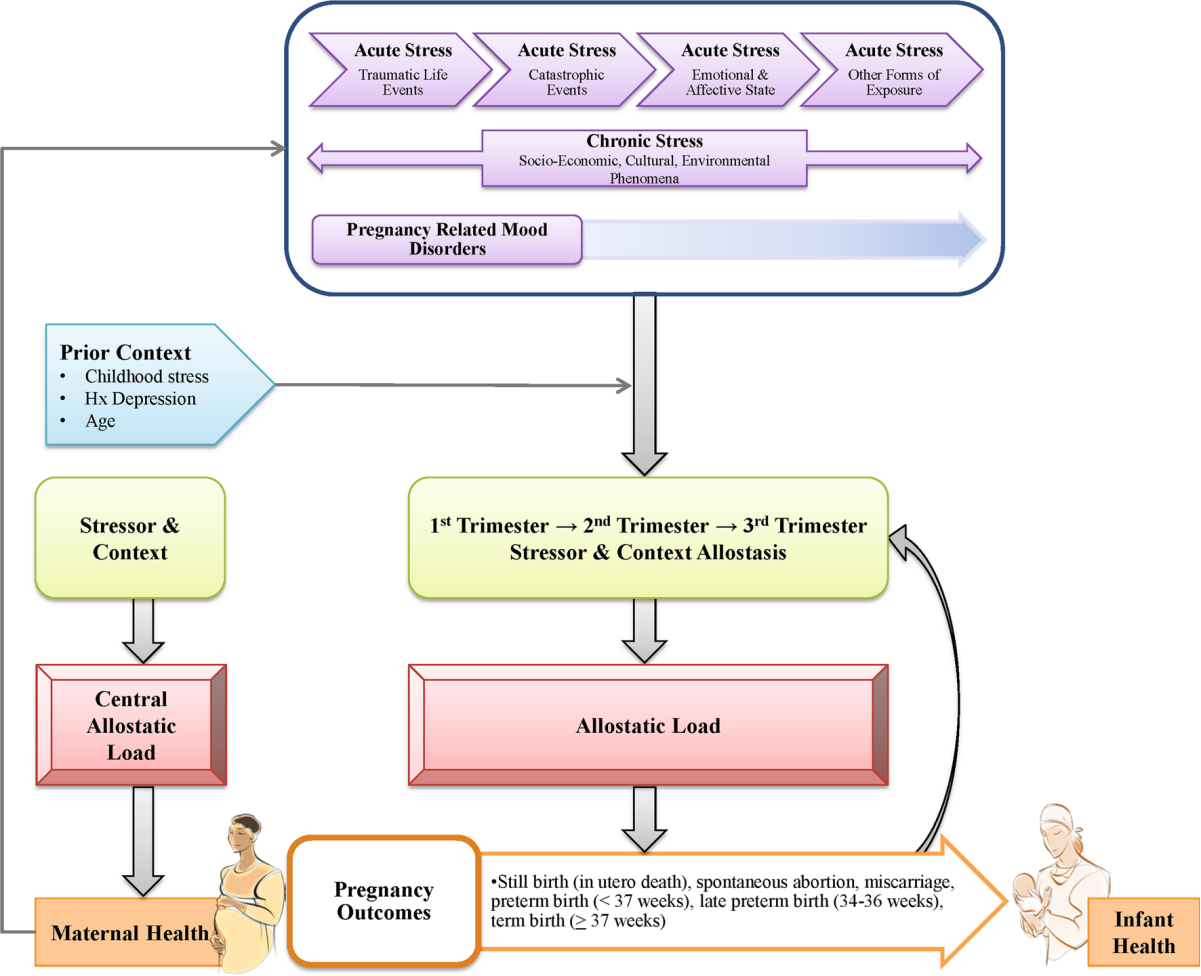
Perinatal distress and pathways to pregnancy outcome: Allostatic load as a conceptual framework

Towards the end of the review, a group of stakeholders (researchers, clinicians, academics, and policy decision-makers) from Pakistan, Kenya, Tanzania, and Canada were brought together to add additional insights and refine the conceptual framework. Terminology, such as perinatal distress, was clarified and a common understanding was developed of concepts. Essential elements of the framework were identified and revisions were made to better illustrate relationships between components. Through an iterative and consensus building process with feedback received from peer-reviewers of this manuscript, we present the final conceptual framework (see Fig. 3).

### Findings

Perinatal mental health of women living in LMIC, particularly mental health during pregnancy, received little attention until 2002. LMIC were represented in only 8 and 15 % of the pregnant- and post-partum related studies, respectively compared to 90 % of high income countries [1, 40]. A systematic review [1] and a report of the World Health Organization-United Nations Population Fund [40] concluded that available literature from LMIC (41 studies) suggests a wide range in prevalence rates of perinatal mental disorders as a consequence of place of recruitment (e.g., tertiary hospital, provincial or district health services, and community facilities), and methodology (e.g., time of data collection, and screening instruments). An average prevalence of 15.9 % (95 % CI 15.0–16.8 %) during pregnancy and 19.8 % (95 % CI 19.2–20.6 %) postpartum were reported [1], with depression and anxiety disorders being the most frequent diagnoses in both periods [1, 40]. The conceptual framework to examine risk factors for perinatal distress varied among the 31 studies and therefore data could not be pooled [1]. Findings related to risk and protective factors of perinatal distress were mixed and the majority did not address all the domains of the social determinants of health used to synthesize the literature, namely, socioeconomic factors, quality of relationship with intimate partner, family and social relationships, reproductive and general health, history of mental health problems, and infant characteristics [1]. Social factors, particularly those beyond the women’s control, seem to influence perinatal mental health of women in LMIC [1]. Moreover, prevention of preterm birth has received little attention in these countries. Although we were able to identify nine studies focusing on preterm birth in LMIC, none examined the relationship between perinatal distress and preterm birth. Five studies [7–10] reviewed epidemiology including global trends, causes, and interventions thus informed this review. However, four studies were excluded as they focused on the use of antenatal steroids in LMIC (two studies), or long-term neurodevelopmental outcomes of preterm infants born in LMIC (two studies). Thus, in LMIC there are missed opportunities to address mental health needs of women along the perinatal continuum and contribute to scientific knowledge and evidence-informed practices and policies to reduce preterm birth and improve infant health outcomes.

### Perinatal Distress Predicts Preterm Birth

The term “perinatal distress” encompasses a spectrum of psychological conditions in response to experiences of episodic and chronic stress associated with adverse socio-economic, cultural, and environmental phenomena [41]. The North American literature suggests that *pregnancy*-*related anxiety*, which relates to the women’s fears about the infant’s health, delivery, her own health and survival during the birthing experience, and the impending responsibility of providing for the child [42], is a stronger determinant of preterm birth than general anxiety [5, 6, 42–44]. Though many North American and European studies have shown an association between *general anxiety* and preterm birth (e.g., [45, 46]), the findings have been mixed (e.g., [47, 48]). In one study, changes in anxiety level over time rather than the anxiety level at one time point predicted preterm birth [49]. North American and European studies examining the relationship between *depression* and preterm birth have also shown inconsistent findings, with a minority of the studies finding a statistically significant association between depression and preterm birth (e.g., [44, 46, 50]).

Many distinguishable forms of stress can be grouped into chronic stressors and episodic (i.e., acute) stressors. *Chronic stress* differs from acute stress, in that the threat or demand is long-lived, and often without resolution [13]. The chronic stress of homelessness or household strain has been associated with preterm birth [41]. A study of 739 low-income African-American pregnant women in the United States found that inadequacy of time and money for non-essentials (e.g., time to look nice, time with friends and family) were mediating factors for preterm birth, whereas multidimensional stress (money worries, family problems, and neighborhood crime) and locus of control were independent predictors of preterm birth [51]. Neighborhood-level stressors, such as poverty, crime, and racial composition have also shown an independent impact on preterm birth [41]. *Episodic stressors* include catastrophic events, such as natural disasters (e.g., hurricane, earthquake, and drought), and manmade calamities (e.g., political strife, and war), have shown varied impacts on pregnancy outcomes from no detected effect (e.g., [52]), to lower [53] and higher [54] rates of preterm birth. The inconsistent findings may be explained by differences in levels of support, medical care, and changes in behavior following the event [41].

Based on the current literature, a multidimensional approach for examining perinatal distress is evident. None of the studies located examined all of the above dimensions of perinatal distress in relation to preterm birth in the same sample. Whether perinatal distress predicts preterm birth in LMIC remains to be established, as none of the studies considered women in LMIC despite nine of the 11 countries with the highest rate of preterm birth being LMIC [9]. In our pilot study [55] the odds of preterm birth were 1.44 times higher in the depressed Pakistani women than in the non-depressed Pakistani women. The social, cultural, and environmental context of LMIC provide the potential for an in-depth investigation of the multidimensional nature of perinatal distress, which could not be achieved in high-income countries, as all dimensions of perinatal distress co-exist in one setting. Furthermore, the void of empirical literature stemming from LMIC on perinatal distress makes it imperative to examine the etiologic contribution of perinatal distress on preterm birth in LMIC.

### Explaining Causal Links of Perinatal Distress to Preterm Birth

In an attempt to adapt or maintain stability (i.e., allostasis), the body responds to perinatal distress (i.e., stress, anxiety, or depression) by producing multisystem physiologic responses through the production of hormonal and neurotransmitter mediators [20, 56, 57]. In addition to being protective or adaptive, these mediators can have damaging effects [58]. Over time, repeated fluctuations and elevated levels of physiologic activity can lead to inefficiency in allostasis where accumulation and overexposure to these mediators (i.e., allostatic load) may results in organ system failure [56, 58]. According to the conceptual framework of allostatic load, composite measures of biomarkers (i.e., hormonal and neurotransmitter mediators) versus individual biomarkers may be a stronger predictor of negative consequences of perinatal distress [56, 59]. The original set of ten parameters of allostatic load continues to expand [60]. Empirically supported allostatic load biomarkers implicated in the pathophysiological process linking perinatal distress to preterm birth include:

#### Cortisol

The brain coordinates the interconnected set of neuroendocrine and behavioral responses to perinatal distress [58, 61]. Cortisol, regulated via the HPA axis, is a primary hormone reported to be elevated in response to stress induced by physical, cognitive and psychosocial challenges [58, 61]. Cortisol is also proposed to be a primary mediator contributing to allostatic load [59, 61]. Although chronically high levels of cortisol have been the focus in the interplay between stress and allostatic load, low cortisol has also been implicated in adverse health outcomes [61]. Consequently, response and recovery promoting optimal functioning of pathophysiologic processes following stress is important when considering allostatis [61]. Thus, low values and high values may be predictive of preterm birth. Cortisol, measured in blood, has been reported in the majority of studies to have a positive association with preterm birth [62].

#### Corticotropin-Releasing Hormone

Pathologic levels of cortisol can increase the production of placental corticotropin-releasing hormone (CRH) in a dose response relationship [63]. Placental CRH levels beyond a certain threshold can have a paradoxical effect of preparing for labor and initiating contractions [63]. In the pregnant state, the diurnal variations in hormones, such as cortisol, are to a certain extent diminished [34]. In an attempt to compensate for the dysregulation of cortisol, systemic responses of the metabolic, inflammatory, and cardiovascular systems may also experience dysregulation [36, 64].

#### Triglyceride, Total Cholesterol, Low-Density Lipoprotein, and High-Density Lipoprotein

Total cholesterol, and high-density lipoprotein (HDL**)**, represent the primary effects in response to dysregulation of cortisol [59]. Hypercholesterolemia (a secondary mediator) may result in response to high levels of cortisol which mobilizes lipids from adipose tissues [65]. Although high levels of cholesterol decreases uterine contractility [65], in combination with the natural lipid profile of pregnancy [66], an allostatic load effect may alter the vulnerability of the uterine smooth muscle thereby changing its propensity to remain quiescent during pregnancy. During pregnancy, the lipid profile of women changes (i.e., increase in triglyceride, total cholesterol, and low-density lipoprotein) in response to hormonal changes occurring with increasing gestational age [66]. Alternation in lipid metabolism, specifically delayed clearance of triglycerides, has been implicated in pregnancy complications (e.g., hypertension and development of preeclampsia) that may lead to medically indicated preterm birth [66], as well as adverse pregnancy and infant outcomes [67].

#### White Blood Cell Count, C-Reactive Protein, and Cytokines

Primary effects, such as changes in inflammatory biomarkers in response to primary neuromediators (i.e., cortisol) of stress, have been implicated in the pathway to preterm birth. A systematic review examining the association between inflammatory cytokines and risk of spontaneous preterm birth in asymptomatic women concluded that the maternal–fetal interface, rather than systemic inflammation, plays a major role [68]. Pregnancy-related anxiety has been associated with preterm birth [6, 69], but among these two studies, only one found that inflammatory markers mediated this influence [69]. Various scales were used to measure pregnancy-related anxiety and samples were drawn from high-income countries with low rates of preterm birth.

Immunosuppression of cellular and humoral immune activity resulting from dysregulation of neuroendocrine mediators, is either site specific (e.g., bacterial vaginosis) [70] or systemic, and may increase risk of infections which may be monitored by examining changes in white blood cells counts. In a meta-analysis, bacterial vaginosis was identified as a strong risk factor for preterm birth, with individual studies repeatedly and consistently demonstrating an association [70]. A connection has also been demonstrated between prenatal stress and C-reactive protein (CRP) [69]. Increased inflammatory cytokines produced both in response to stress (primary mediators) and in response to the infection stimulates production of CRP and triggers prostaglandin production which is a mediator of labor [71]. Typically increased cortisol levels serve as a negative feedback loop to decrease production of cytokines and hormones [71]; however, the dysregulation of neuromediators most likely impairs this negative feedback loop.

#### Immunoglobulin G

Immunoglobulin G, an antibody that crosses the placenta, is critical in protecting the infant from infection in the neonatal period. Lower transplacental ratios of immunoglobulin G have been reported in preterm infants [72]. High levels of immunoglobulin G, in response to dysregulation of cortisol, is proposed to saturate binding sites, thereby limiting the placenta’s efficiency in transfer of immunoglobulin G [73]. Since the infant’s humoral response is inefficient, the impaired transfer of immunoglobulin G may further compromise the infant’s ability to fight infection in early life [73] and increase risk of mortality.

#### Blood Pressure and Heart Rate

Increased blood pressure and heart rate represent a disease state or disorders resulting from allostatic load, as a consequence of secondary outcomes and primary mediator of stress [59]. Cardiovascular reactivity is normally reduced in pregnancy [74]. However, increased levels of cortisol may increase maternal cardiovascular reactivity (e.g., increase blood pressure and heart rate—secondary mediators) [34] by altering maternal, placental or fetal hemodynamics [75]. A relationship has been demonstrated between high diastolic blood pressure responses to stress during pregnancy and decreased gestational age at birth [75–78]. A dose–response pattern has been observed between the rise in blood pressure and spontaneous preterm birth [79].

There is empirical support (approximately 60 studies) for an association between increased allostatic load and negative health consequences of stress (e.g., cardiovascular disease) [80]. Notably, none of the documented studies (e.g., [6, 55, 81–85]) examining the relationship between perinatal distress, biomarkers of stress, and preterm birth have made use of allostatic load in their conceptual framework. Moreover, the scales used to measure perinatal distress, biomarkers of stress examined, time periods of measurements and findings have varied between studies (see Table 2). Individual mediators of stress examined in these studies included cytokines (interleukin-10, interleukin-6 and tumor necrosis factor-alpha), CRP [69], cortisol [6], and CRH [6]. Interrelated physiological (i.e., biochemical) response patterns [86, 87] and composite measures involving several biochemical measures offer a more objective and quantifiable indicator of the level of perinatal distress in pregnant women in LMIC who are in difficult cultures, than self-report psychological measures of perinatal distress [56, 59]. The risk of preterm birth will be higher when there is an inadequate response to prenatal distress (i.e., high perinatal distress and low allostatic load) or prolonged response to a previous stress (i.e., low perinatal distress and high allostatic load) [20, 57]. Identifying high risk pregnant women in LMIC and understanding the pathophysiological process of poor pregnancy and health outcomes will guide the development and evaluation of therapeutic interventions to avert preterm birth.

**Table 2:** Summary of studies examining the relationship between prenatal stress, biomarkers of stress, and preterm birth

Study and country (region)	Design	Participants (n)	Measures			Results
			Scales	Specimen	Time, gestation (weeks)	
Hobel et al. [81] USA (Los Angeles)	Prospective case–control study^a^	Subsample of 524 Cases: 18 (spontaneous onset of preterm labor) Control: 18 (delivered at term) Inclusion/exclusion criteria: not specified	PSS-8; STAIT-10	Plasma CRH, ACTH, cortisol	18–20 28–30 35–	Higher plasma CRH levels and ACTH levels were reported at all three time periods and elevated cortisol levels at 18–20 weeks’ gestation and 28–30 weeks’ gestation in women who delivered preterm when compared to those who delivered at term. Stress levels did not differ between 18–20 weeks’ gestation and 28–30 weeks’ gestation. Variance in CRH at 28–30 weeks’ gestation was explained by maternal stress level at 18–20 weeks’ gestation and maternal age.
Erickson et al. [82] Denmark (Odense)	Prospective case–control cohort design^a^	Subsample of 2,927 Cases: 84 (delivered preterm [idiopathic etiology] without complications) Control: 224 (delivered at term and matched, at time of enrollment, to within 10 days of due date of cases) Inclusion criteria: age >18 years, ability to understand Danish. Exclusion criteria: insufficient responses to the questionnaires, placental previa (diagnosed after 30 full gestational weeks), history of severe fetal congenital malformations in previous pregnancy, uterine cervix insufficiency treated with cervical circlage	Three questionnaires: (1) just before inclusion (past medical history); (2) 30 weeks’ gestation (social and demographic information); (3) birth; (urogenital and obstetric problems) If delivered preterm, completed second and third questionnaire at same time	Plasma CRH, CRH-binding protein, cortisol Venous blood sample taken during labor (delivered preterm), and 37–43 weeks’ gestation (delivered at term)	7–23 27–37	7–23 weeks: CRH and CRH-binding protein levels were higher in women who delivered preterm when compared to women who delivered at term. 27–37 weeks’ gestation: CRH and cortisol levels were higher but CRH-binding protein levels were lower in women who delivered preterm when compared to women who delivered at term. Previous preterm delivery and engagement in some risk-taking behaviors were associated with preterm birth
Ruiz et al. [83] USA (central Texas)	Prospective, longitudinal, observational study	Cases: 78 Inclusion: English speaking, <28 weeks’ gestational age, 18–40 years of age, singleton pregnancy. Exclusion criteria: Rh isoimmunization, cervical cerclage, use of tocolytic agents during current pregnancy, diabetes mellitus requiring insulin, thyroid disorders, chronic renal or heart disease, misses more than 1 monthly prenatal check for data collection	PSS-10 (23–26, and 31–35 weeks’ gestation)	Blood cortisol (all time points); vaginal swabs for fetal fibronectin, chlamydia, and bacterial vaginosis screen (23–26 and 27–30 weeks’ gestation)	15–19 20–22 23–26 27–30 31–35	Cortisol was a poor predictor of both preterm labor and preterm birth; however an increase in cortisol level was noted in women with genitourinary infection. Change is PSS score, that is decrease in perceived stress during the 2nd trimester, was significantly associated with increase in length of gestation
Mancuso et al. [84] USA (Los Angeles)	Case–control study nested in a prospective cohort^a^	Subsample of 688 Cases: 282 Inclusion criteria: singleton intrauterine pregnancy, gave birth to liveborn infant, received prenatal care in prenatal clinics and private practices in Los Angeles, California. Exclusion criteria: age <18 years, stillborn births, multiple gestation births, lack of birth outcome data, and incomplete psychosocial data	PSA	Plasma CRH	18–20 28–30	Women with high CRH levels and high maternal prenatal anxiety at 28–30 weeks gestation delivered earlier. CRH levels were significantly higher at both times points in women delivered preterm than women who delivered at term. Mediation effect of CRH
Kramer et al. [6] Canada (Montreal)	Prospective cohort and nested case–control design	Subsample of a larger study Cases: 207 Control: 444 Inclusion criteria: age ≥18 years, singleton gestation, and able to speak English or French. Exclusion criteria: severe chronic illness with ongoing treatment (note: other than hypertension, asthma, or diabetes), placenta previa, diagnosis of incompetent cervix in previous pregnancy, impending delivery, or fetus with congenital anomaly	DHS (lacked basic or essential needs subscale), MSS (chronic stress), AAS (conjugal violence), 5-item scale (injury, job related stress), MIS (intention of pregnancy), ASSIS (perceived social support), PLES (acute stressors), PSS, Dunkel-Schetter 4-item scale (pregnancy related anxiety), RSES, LOT (optimism and pessimism), CES-D, single item (woman’s perception of her risk of birth complications), 8-item scale (commitment to pregnancy)	Hair cortisol, histo-pathologic examination of vaginal swabs, placenta, and cord	24–26	Only pregnancy related anxiety was consistently and independently associated with spontaneous preterm birth and a dose–response was reported across quartiles. Hair cortisol was positively associated with gestational age but not CRH. Maternal plasma CRH, hair cortisol, placental histopathology (i.e., features of infection/inflammation, infarction, or maternal vasculopathy) were not associated with stress, anxiety, or distress measures
Pearce et al. [85] Denmark (Odense)	Case–control study nested in a prospective cohort	Subsample of 2,927 Cases: 60 [delivering preterm (<37 weeks) without a cause, as determined from clinical findings or laboratory investigations during pregnancy or at delivery] Control: 123 (delivering at term) Inclusion criteria: age >18 years, ability to understand Danish. Exclusion criteria: insufficient responses to the questionnaires, placental previa (diagnosed after 30 full gestational weeks), history of severe fetal congenital malformations in previous pregnancy, uterine cervix insufficiency treated with cervical circlage	Questionnaire (stressful life events, risk-taking behavior indicated by lack of seat-belt usage)	Serum measures of cortisol, MIF, CRP, CRH, interleukin-1 ß, interleukin-6, interleukin-10, tumor necrosis factor-alpha	<24	Individual biomarkers: MIF (strongest association), interleukin-10, CRP and tumor necrosis factor-alpha predicted preterm birth at various cutoff levels (e.g., 75th, 85th, and 90th percentile). Logistic regression models: MIF, CRP, risk-taking behavior, and low education consistently predicted preterm birth at various cutoffs; however, the 75th percentile cutoff was the best predictive model. MIF may be a psychobiological mediator
Shaikh et al. [55] Pakistan (Kirachi)	Prospective cohort study design	Cases: 132 (125 with complete data) Inclusion criteria: age 18–40 years, 28–30 weeks’ gestation. Exclusion criteria: diabetes mellitus, thyroid disorder, chronic renal or heart disease, or uterine and cervical abnormality, or on antidepressants or other psychotropic drugs, and did not deliver in setting where the study was based	A–Z Stress Scale, CES-D	Serum cortisol	28	A significant positive relationship was reported between maternal depression and stress. No relationship was noted between cortisol value and stress scale or depression scale. Preterm birth was associated with higher parity, past delivery of a male infant, and higher levels of paternal education

Adopted from Shaikh et al. [39]

*AAS* Abuse Assessment Screen (adapted), *ACTH* adrenocorticotropic hormone, *ASSIS* Arizona Social Support Interview Schedule, *CES*-*D* Centre for Epidemiology Studies Depression Scale, *CRH* corticotropin-releasing hormone, *CRP* C-reactive protein, *DHS* Daily Hassles Scale, *LOT* Life Orientation Test, *MIF* macrophage migration inhibitory factor, *MIS* Miller Intendedness Scale, *MSS* Marital Strain Scale of Pearlin and Schooler, *PLES* Prenatal Life Events Scale, *PSA* Pregnancy-Specific Anxiety Scale, *PSS*-*8* Perceived Stress Scale 8-item version, *PSS*-*10* Perceived Stress Scale 10-item version, *RSES* Rosenberg Self-Esteem Scale, *STAIT* Spielberger’s State Anxiety Inventory 10-item version

^a^Not labelled

### Explaining Causal Links of Perinatal Distress to Infant Health

Allostatic load or overload exerts its influence on biological indices or mediators of the HPA axis and sympathetic–adrenal–medullary systems involving a complex interplay between the mother and fetus [13, 59]. The dysregulation of cortisol influences the permeability of the placenta to cortisol, thereby altering the placental and fetal environment [64] and potentially increasing permeability of other mediators which typically do not cross the placenta (e.g., epinephrine). The health of the fetus and newborn “mirror” the health of the mother whereby the fetus or newborn mimics the biochemical profile of the mother. Allostatic load in the fetal brain may also alter behavioral systems which involve attachment/approach and avoidance behaviors that are integral to survival [16, 18, 58]. Allostatic load may also alter the function (e.g., affective, cognitive, and social) and structure of the brain, and pathological levels may impact developmental outcomes [19]. Perinatal distress may directly (e.g., alter structure and function of brain) or indirectly (i.e., through mother–infant interaction) influence infant health and well-being.

In addition to activating the HPA axis, and sympathetic, immune and cardiovascular systems, psychosocial health during pregnancy has been linked to negative maternal health behavior (e.g., consuming non-nutritive substances like soil, consumption of alcohol, and cigarette smoking) [88]. Maternal prenatal distress and postnatal distress may result in the same disorders simply manifested along the perinatal continuum [89]. Altered parenting patterns (i.e., lack of responsivity to infants’ needs [90, 91], inability to coordinate age-appropriate activities [92], and harsh parenting style [93] ) observed in mothers with PPD may contribute to infant stress, with cumulative stress influencing vulnerability to death, disease, or poor developmental outcomes through the effects of infant allostatic load [15–21]. Although in LMIC there is limited evidence examining the contribution of prenatal distress to infant health outcomes (e.g., [94]), there is extensive scientific evidence linking PPD and infant health [95] that may be explained by the conceptual framework of allostatic load.

Our pilot data suggests that the odds of depression are 2.7 times greater (95 % CI 1.16–6.17, *p* = 0.015) in Pakistani mothers of preterm infants than Pakistani mothers of full-term infants [96]. Thus, for infants born in LMIC, the interactive effects of biological vulnerability associated with being born premature, social vulnerability inherent in women’s responses to their environment during the postpartum period and inequities in determinants of health (i.e., poverty, poor nutrition) places them at triple jeopardy to experience poorer health outcomes. In LMIC, premature infants’ chance of survival, well-being and lifetime developmental and behavioral success may depend on reducing or managing risk factors associated with perinatal distress. For example, implementing early interventions to reduce the risk of stress, anxiety or depression during pregnancy or improve maternal behavior (i.e., increase responsiveness to infant) in the months following the birth of the infant may be warranted.

## Discussion

The conceptual framework of allostatic load relates preterm birth to the social, environmental, and biological antecedent of perinatal distress, thereby enabling researchers to examine the interrelationships between various determinants of health. It provides an integrated model that is essential to examine the nature of risk (i.e., cumulative risk) across many systems at the same time and the temporal effects of the risk(s). The use of the conceptual framework of allostatic load to examine the etiologic contributions of perinatal distress on pregnancy and infant outcomes will necessitate longitudinal study designs with multiple time points (e.g., first trimester, early and late second trimester, and third trimester), and multiple measures of data collection (i.e., all dimensions of perinatal distress).

Although for our purpose we have focused on the negative pregnancy outcome of preterm birth, the conceptual model can be used to investigate pathways for positive pregnancy outcomes. A positive health focus may facilitate population level interventions directed at promoting mental health during pregnancy or “salutogenesis” within the context of their social, cultural, and political environment [97]. In LMIC, focusing on what makes women resilient in the face of toxic stress (i.e., pervasive, uncontrollable stress)—that is, improving their sense of coherence or “way of being in the world” [98]—may reduce the burden of health care service delivery. Furthermore, this type of research will facilitate identification of culture-sensitive strategies [98] to promote the mental health of women along the perinatal continuum. However, it will be important to debate and discuss social and cultural norms and policies that undermine, both at an individual level and society level, women’s mental health during pregnancy and postpartum and access to mental health services.

Building research capacity will be essential to addressing the under-representation of pregnancy and post-partum related studies in LMIC. Facilitating partnerships between researchers in high-income countries and LMIC to identify and resolve unique challenges related to ethical conduct of research will be important to generating new knowledge in LMIC. Key among these challenges is the communication and understanding of informed consent [99]. Since women in LMIC are underprivileged (e.g., poor, with limited access to health care), they may be particularly vulnerable to coercion. Moreover, in keeping with the World Medical Association Declaration of Helsinki, the research should “be responsive to the health needs and priorities of this population or community” [100]. Since mental health care services may be non-existent or limited and predominantly hospital based [101] consideration should be given to developing or strengthening local mental health care referral services that will continue to serve the women after completion of the study. Strategies (e.g., referrals) will need to be developed to minimize risk and prevent harm to women participating in the study over the course of their pregnancy and following birth of their baby.

Aside from these ethical issues, studies involving blood sampling for allostatic load parameters need to critically consider the available laboratory infrastructure. Study procedures including procurement of laboratory samples, storage, transportation and processing may create technical and logistical difficulties. Establishing standard procedures, training and supervision of local researchers to develop research capacity, and assisting with knowledge transfer may mitigate logistical issues and ensure adherence to study protocols [102, 103]. Furthermore, quality assurance measures may need to be established to ensure quality data [104]. Recruitment and retention of subjects may present significant challenges [105] as infrastructure, including communication to arrange follow-up visits and clinical facilities for care may be lacking [102].

## Conclusion

Pregnant women in LMIC have been a neglected population in studies on perinatal distress and pregnancy and infant outcomes. Given inequities in determinants of health and the social, cultural, and political contexts of childbearing women in LMIC, these women may experience differential vulnerability to risk factors for perinatal distress and poor pregnancy outcomes. Prospective studies with multiple biological and psychosocial measures of stress, depression or depressive symptoms and its antecedents (e.g., childhood stress, major life events, etc.), state and trait anxiety, and pregnancy-related anxiety may add new knowledge and enhance our understanding about the etiologic contributions of psychosocial processes to preterm birth. A theoretical framework of allostatic load will enable researchers to concurrently examine social, environmental, and genetic antecedents of stress-related vulnerability and physiological (e.g., immune system, placenta) and behavioral responses that influence not only pregnancy outcomes of women in LMIC but also the life trajectories of health and wellness of the fetuses/infants (i.e., mortality and morbidity over time) [64]. Interrelated physiological (i.e., biochemical) response patterns [86, 87] and composite measures involving several biochemical measures offer a more objective and quantifiable indicator of the level of perinatal distress in pregnant women in LMIC. We propose that researcher maintain a positive health focus by identifying protective factors or processes that contribute to resilience in the face of toxic stress. When planning research studies using an integrative approach with both biological and psychosocial measures in LMIC, of critical importance is the adherence to principles of ethical conduct of research, engaging local researchers and other stakeholders to anticipate operational challenges to conducting research, and ensuring that the research is responsive to the needs of women during the perinatal period.
